# Comparing conventional and Bayesian workflows for clinical outcome prediction modelling with an exemplar cohort study of severe COVID-19 infection incorporating clinical biomarker test results

**DOI:** 10.1186/s12911-025-02955-3

**Published:** 2025-03-10

**Authors:** Brian Sullivan, Edward Barker, Louis MacGregor, Leo Gorman, Philip Williams, Ranjeet Bhamber, Matt Thomas, Stefan Gurney, Catherine Hyams, Alastair Whiteway, Jennifer A. Cooper, Chris McWilliams, Katy Turner, Andrew W. Dowsey, Mahableshwar Albur

**Affiliations:** 1 Department of Population Health Sciences, Bristol Medical School, University of Bristol, Bristol, UK; 2 Department of Engineering Mathematics, Faculty of Engineering, University of Bristol, Bristol, UK; 3 Department of Microbiology, University Hospitals Bristol and Weston NHS Foundation Trust, Bristol, UK; 4 Intensive Care Unit, University Hospitals Bristol and Weston NHS Foundation Trust, Bristol, UK; 5 Severn Infection Sciences, Southmead Hospital, North Bristol NHS Trust, Bristol, UK; 6 Department of Clinical Heamatology, Southmead Hospital, North Bristol NHS Trust, Bristol, UK; 7 Intensive Care Unit, Southmead Hospital, North Bristol NHS Trust, Bristol, UK; 8 Jean Golding Institute, University of Bristol, Bristol, UK

**Keywords:** Projective prediction, Bayesian, Logistic regression, Risk factors, COVID-19

## Abstract

**Purpose:**

Assessing risk factors and creating prediction models from real-world medical data is challenging, requiring numerous modelling decisions with clinical guidance. Logistic regression is a common model for such studies, for which we advocate the use of Bayesian methods that can jointly deliver probabilistic risk factor inference and prediction. As an exemplar, we compare Bayesian logistic regression with horseshoe priors and Projective Prediction variable selection with the established frequentist LASSO approach, to predict severe COVID-19 outcomes (death or ICU admittance) from demographic and laboratory biomarker data. Our study serves as guidance on data curation, variable selection, and performance assessment with cross-validation.

**Methods:**

Our source data is based on a retrospective observational cohort design with records from three National Health Service (NHS) Trusts in southwest England, UK. Models were fit to predict severe outcomes within 28 days after admission to hospital (or a positive PCR result if already admitted) using demographic data and the first result from 30 biomarker tests collected within 3 days after admission (or testing positive if already admitted).

**Results:**

Patients included hospitalized adults positive for COVID-19 from March to October 2020, 756 total patients: Mean age 71, 45% female, 31% (n=234) had a severe outcome, of whom 88% (n=206) died. Patients were split into training (n=534) and external validation groups (n=222). Using our Bayesian pipeline, we show a reduced variable model using Age, Urea, Prothrombin time (PT) C-reactive protein (CRP), and Neutrophil-Lymphocyte ratio (NLR) has better predictive performance (median external AUC: 0.71, 95% Quantile [0.7, 0.72]) relative to a GLM using all variables (external AUC: 0.67 [0.63, 0.71]).

**Conclusion:**

Urea, PT, CRP, and NLR have been highlighted by other studies, and respectively suggest that hypovolemia, derangement of circulation via clotting, and inflammation are strong predictive risk factors of severity. This study provides guidance on conventional and Bayesian regression and prediction modelling with complex clinical data.

**Supplementary Information:**

The online version contains supplementary material available at 10.1186/s12911-025-02955-3.

## Introduction

Estimating predictive risk factors for disease outcomes with explainable statistical models is desirable for clinical use and decision making. Clinical resources are typically limited, and variable selection techniques that can reduce complex multivariate models to ones with a smaller subset are useful as they can offer similar performance without the resource cost of collecting additional test results. We provide a guide for modern Bayesian approaches for joint risk factor analysis and variable selection demonstrated in a patient dataset obtained from UK hospitals during the first wave of the COVID-19 pandemic.

We analyze a range of laboratory blood marker values across metabolic pathways affected by COVID-19 infection and evaluate predictive models of severe outcomes. We: (a) Examine statistical associations of routinely measured blood biomarkers, and age and gender, to predict severe COVID-19 outcomes; (b) Develop cross-validated logistic regression prediction models using the candidate biomarkers, highlighting biomarkers worthy of future research. (c) Employ variable selection techniques, comparing the least absolute shrinkage and selection operator (LASSO) frequentist method [1] to the recent Projective Prediction approach [2] on Bayesian logistic regression models with horseshoe priors to illustrate the process of creating a reduced model that maintains similar performance while being more feasible to implement clinically; (d) We demonstrate a balance between best analytic practices and pragmatic solutions for clinical data curation and statistical modelling decisions emphasizing the benefits of the proposed Bayesian workflow.

While our paper is methodological, we detail several aspects of COVID-19 and surrounding research to motivate circumstances around the dataset we obtained and the types of clinical decisions made. Further, clinical considerations motivate why variable selection can play a crucial factor in modeling. Globally, COVID-19 has resulted in hundreds of millions of cases and millions of deaths (WHO Coronavirus (COVID-19) Dashboard https://covid19.who.int/). COVID-19 has a wide spectrum of clinical features ranging from asymptomatic to severe systemic illness with a significant attributable mortality, while clinical manifestations are variable especially in the most vulnerable groups and immunocompromised people [3]. COVID-19 is a multi-system disease resulting in the derangements of homeostasis affecting pulmonary, cardiovascular, coagulation, haematological, oxygenation, hepatic, renal and fluid balance [4–6]. During the first wave of the pandemic, the majority of people with COVID-19 had mild or no symptoms, but an estimate of one in five to one in 10 needed hospitalisation [7]. Early identification of hospitalised COVID-19 patients who are likely to deteriorate, i.e. transfer to ICU or who may die, is vital for clinical decision making.

Several prediction models have evaluated case-level factors that might predict poor outcomes (critical illness or death). A recent living systematic review [8] identified 265 prognostic models for mortality and 84 for progression to severe or critical state. The majority of the studies looked at vital signs, age, comorbidities, and radiological features. According to the review, models were unlikely to include a broad range of variables concerning co-infection, biochemical factors (outside of C-reactive protein), and other haematological factors on an individual patient level. Further, most prognostic models did not describe the target population or care setting adequately, did not fully describe the regression equation, showed high or unclear risk of bias and/or were inadequately evaluated for performance. These drawbacks highlight a need to demonstrate sound practices for severity prediction modeling. Collins et al. and Riley et al. have written a compelling series of such recommendations on many shortcomings of clinical prediction models and steps to remedy [9–11].

We compiled a COVID-19 dataset that is novel in the broad number of blood biomarkers included from clinical laboratory testing, supported by routine patient demographic information. Our dataset was captured with the intent to create a clinical severity score to complement those using physiological data for use during the pandemic, but it became apparent that the dataset was not adequately powered to definitively answer this question. We deviate from suggestions from Riley et al. and Collins et al. concerning sample sizes for data as our dataset is limited in the number of severe outcome examples. We emphasize that the work here is primarily a guide and not intended to make a definitive statement for COVID-19 prediction models. The experiences gained over the course of this research led us to refocus our attention on demonstrating our statistical workflows for this complex data. We highlight two methodologically sound contemporary models from Knight et al. and Carr et al. [12, 13] with better powered studies, but neither record the same biomarkers as each other or our dataset (making direct comparison difficult), nor do they use Bayesian approaches for modeling or variable selection, the present work’s strength.

## Methods

### Overview

Using complex clinical data, we use logistic regression to predict the likelihood of a severe outcome (death or transfer to ICU) for a patient based on demographic information and any available patient biomarker data collected during a 3-day time window starting from being admitted to hospital with COVID-19 or testing positive if already admitted. If a patient transferred to ICU during the 3-day window, we only consider data collected prior to transfer. We highlight the benefits of a Bayesian approach and then focus on variable selection. Clinicians must balance time, money, and equipment access all while trying to deliver high quality patient care. Models that deliver good prediction performance with a small amount of biomarker data are valued for their efficiency.

### Study cohort and demographics

Following a retrospective observational cohort design, anonymized data were obtained from Laboratory Information Management Systems (LIMS) linking patient data for laboratory markers to key clinical outcomes. Three hospitals in the Southwest region of England, UK, participated in the study, two of which were tertiary teaching hospitals and the third was a district general hospital (DGH).

The study underwent a rigorous ethical and regulatory approval process, following an Integrated Research Application System application [IRAS project ID: 283439], a favourable written authorization was gained from NHS Research Ethics Service, Wales Research Ethics Committee 7, c/o Public Health Wales, Building 1, Jobswell Road, St David’s Park, SA31 3HB on 11/09/2020. Our research complies with the declaration of Helsinki with anonymized data and ethical review, as explained below informed consent for data sharing was waived due to overriding public interest. See: https://www.hra.nhs.uk/about-us/committees-and-services/res-and-recs/

The requirement for informed consent was waived by NHS Research Ethics Service, Wales Research Ethics Committee 7 (see above), given overriding public interest in the research. Furthermore, during project development prior to ethics review, a public and patient involvement meeting conducted at North Bristol NHS Trust by author MH received similar support. North Bristol NHS Trust and University Hospitals Bristol and Weston NHS Foundation Trust signed data sharing agreements confirming this waiver. See: https://www.england.nhs.uk/publication/information-sharing-policy All data were fully anonymized before they were transferred to the research team for analysis.

A system-wide data search was conducted on the LIMS for all patients who tested positive for SARS-CoV-2 by polymerase chain reaction (PCR) at these three hospitals during the first wave of COVID-19 pandemic. Data were collected from records between March 1, 2020 to October 31, 2020, with research data access authorized from January 1, 2021 to present day. Serial laboratory data collected as a part of standard of care of patients admitted with/for COVID-19 were included: bacteriology, virology, mycology, haematology, and biochemistry. All patients testing negative for SARS CoV-2 by PCR were excluded. All laboratory markers including clinical outcomes from LIMS were extracted and the final dataset was anonymized with no patient identifying data to link back.

### Inclusion and exclusion criteria

**Fig. 1:**
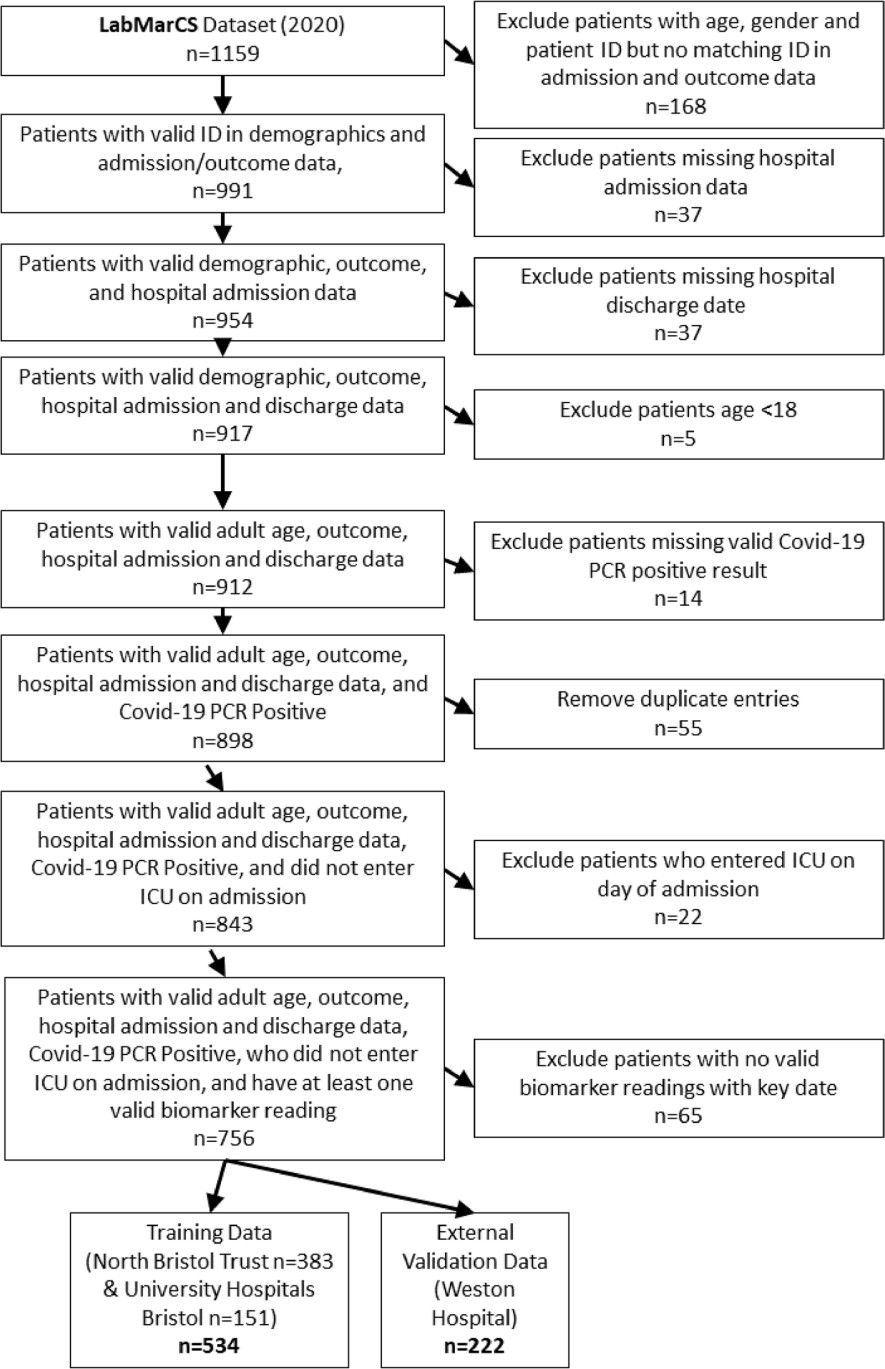
Flowchart of patient exclusion and inclusion criteria. The initial set of 1159 candidate patients was narrowed to a training set (n=534) and an external validation set (n=222)

To be included in the study we had several mandatory criteria. We included all adult patients admitted to the study’s hospitals between March to October 2020 and tested positive for SARS CoV-2 by PCR. Pediatric patients (<18 years old) were excluded. Hospital staff/healthcare workers and their house-hold contacts were excluded prior to data transfer (as it was marked on COVID-19 test requests). Figure 1 depicts the decision flow for inclusion and exclusion of patient data. Furthermore, all patients required age, gender, complete admission/discharge records, and records of their outcome with COVID-19. If a patient had multiple admissions, only the most recent admission since a positive COVID-19 test was considered. Despite our data request constraints, the data transferred contained records outside our criteria. For example, not all patients had records indicating a positive COVID-19 test; we speculate there was a data processing or human error. When combined with restrictions on biomarker data, this considerably narrowed our data set from 1159 patients to 736 who met all criteria, as detailed in the flow chart.

### Predictors (data covariates)

Our dataset includes a variety of clinical severity indices, microbiological, immunological, haematological and biochemistry parameters used as predictive variables in the regression models. A full list of recorded data items is shown in Table 1

**Table 1:**
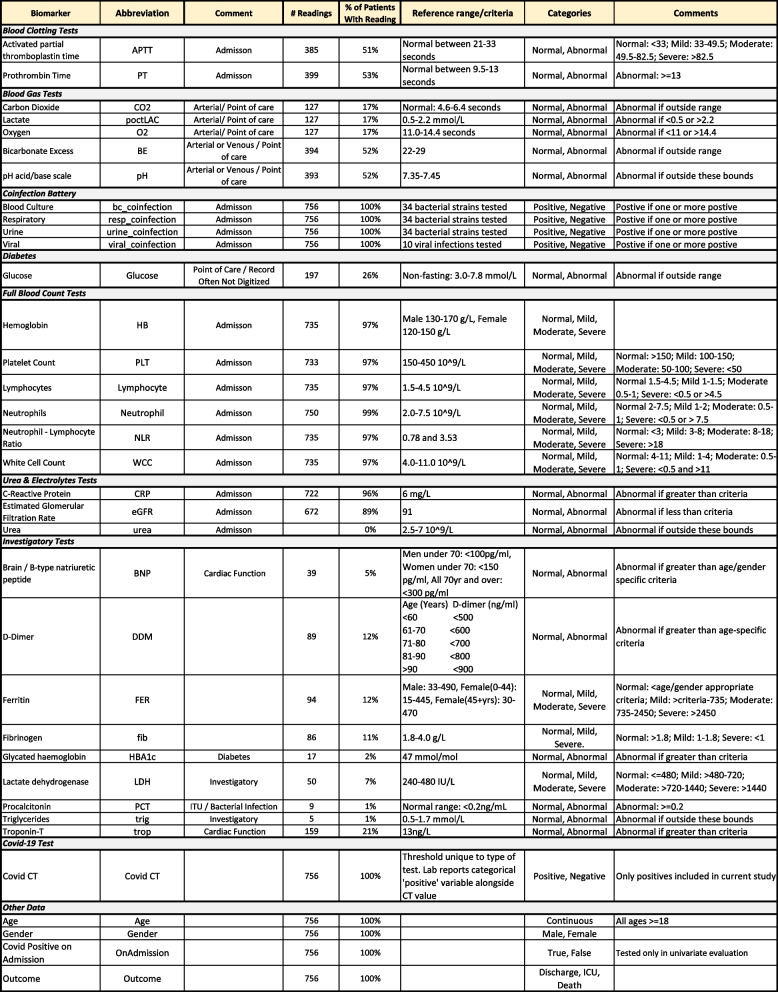
Variables recorded in the study dataset, including plain text description, abbreviation, place of record, frequency in the dataset, and criteria used for converting continuous readings into categorical values

### Outcomes

For all sites, the primary prediction outcome was death or transfer to the ICU within 28 days after the key date. This key date was either the point of admission to hospital, or the date of the first positive COVID-19 PCR test result if the patient was already admitted. 28 days was chosen due to clinical convention and advice from our clinical colleagues, using a different time window may be justified in other circumstances. The distribution of severe outcomes after the key date is right skewed, with 75% occurring within 10 days, with a mean of 7.6 days and standard deviation of 5.5.

### Patient timelines

**Fig. 2:**
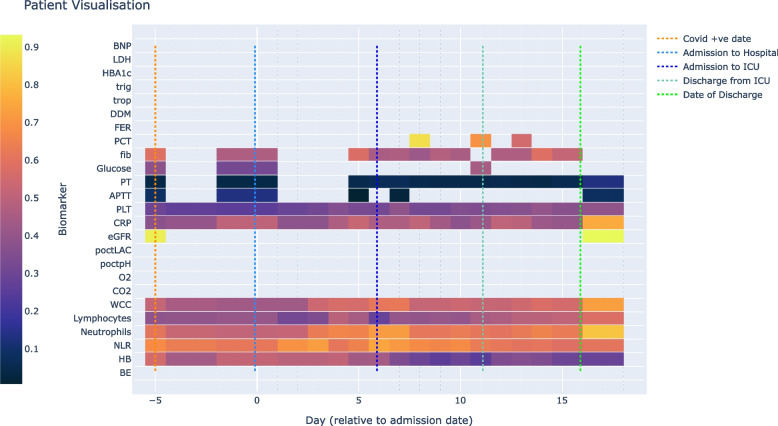
Example of a single patient’s time series laboratory biomarker data. Covid +ve indicates the time of a COVID-19 positive test. See Table 1 for biomarker abbreviations. Biomarkers vary widely in units of measurement. As a simple indication of upward and downward movement of readings, variation in biomarker measures are visualised from low (purple/black) to high (orange/yellow) created by subtracting the minimum value and normalizing the readings to span 0 to 1

The collected laboratory biomarkers are continuous measures and provide a time-series representation of the course of a patient’s admission. Figure 2 shows an example of a single patient’s readings over the course of 18 days between testing positive for COVID-19 and being released from hospital care. This provides a representative example of the heterogeneity seen in our dataset, i.e. not all tests are taken and others are taken regularly or intermittently (further examples in Supplementary Materials A2-A6).

### Transformation of biomarker data

Prediction modelling of irregularly sampled time-series data is a challenging open research question [14]. In this study we focused on established and available tools for conventional and Bayesian prediction. To balance inclusion of biomarker data not available on the day of admission and the need for clinical decisions to be guided soon after admission, we chose to consider the first value recorded for each biomarker within three days after their ‘key date’. We additionally considered the worst or best readings within 1, 5 or 7 days after the key date, and found the first reading within 3 days after the key date to offer a reasonable compromise between prediction performance and speed to inform decision making. Systematic exploration of these parameters would be worthwhile to optimize performance in coordination with clinical needs, but is the beyond the scope of this paper.

**Fig. 3:**
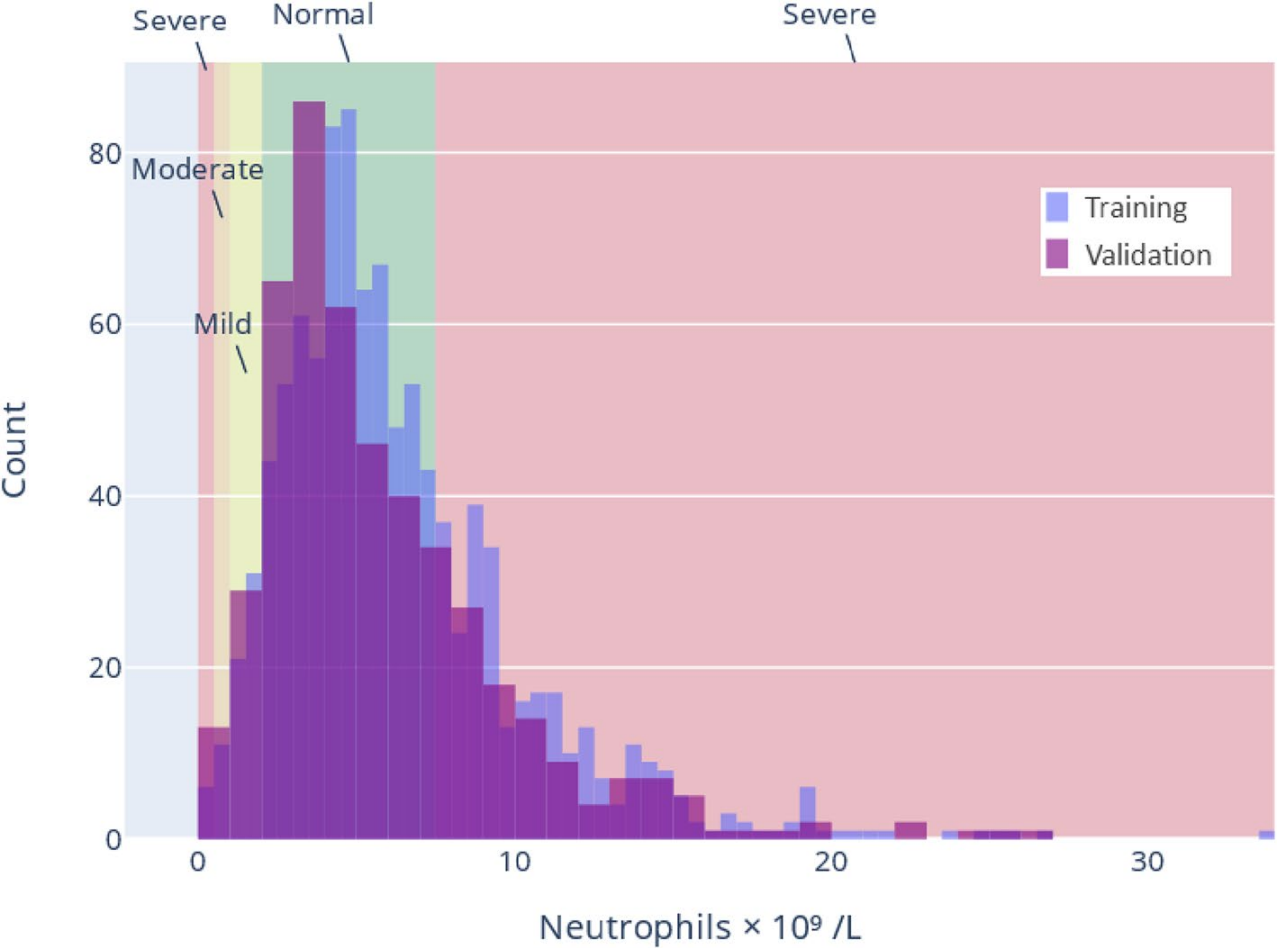
Example distribution of biomarker readings for Neutrophil training and external validation data. Vertical lines indicate clinical thresholds for bounds on Normal, Mild, Moderate, and Severe categorization

In addition, we transformed continuous biomarkers into categorical variables via reference ranges for clinical use in the typical healthy population ranges, see Table 1. These categories are actively in use at laboratories at the participating trusts and were arrived at through a combination of clinician advice, handbooks [15], and guidance from lab test manufacturers. Such transforms are not a trivial decision and there are merits to both hand-crafted transforms informed by domain experts (as we have chosen) versus data driven approaches. On one hand, clinical experience has delineated useful categories of biomarker readings, but it is not evident a priori that such categories are removing nuance present in a continuous measure, especially in the case of a novel disease. Furthermore, a transform could be learned across studies or tuned for a particular dataset. However, this requires sufficient representative data and may require further choices of transformation or non-linear modelling approaches. As an example, Fig. 3 shows the histogram of readings for all values recorded for Neutrophils, including clinical thresholds to transform into categorical data. No missing data imputation was performed, instead missingness was coded as as an additional category ‘Test not taken’. The distribution of how many patients fell into each category per biomarker can be found in Table 2

For further elaboration of these modelling choices and the challenges, please see the Discussion.

## Statistical analysis

Analytics were carried out using the R statistical language (v4.4.1) and R Studio (2024.09.0). We used the following packages: Standard logistic regression analyses used the R Stats GLM package (v4.4.1); LASSO analyses, GLMnet (v4.1.8); and for Bayesian analyses, BRMS (v2.22.0) and ProjPred (v2.8.0). Source code for this analysis pipeline can be found at https://github.com/biospi/LABMARCS.

### Analysis of individual biomarkers

Before running full regression models, we examined the independent contribution of individual biomarkers in the training dataset predicting ICU entry or death via standard logistic regressions and Bayesian logistic regressions with either a flat (aka uniform) or horseshoe prior. This allowed calculation of *p*-values and odds ratios for each biomarker. A 5-fold cross-validation (repeated 20 times was run for each biomarker to estimate median AUC and 95% interquartile intervals. Stratified cross-validation data shuffling was pre-computed per biomarker so models used the same starting data. When performing basic cross-validation, it is possible that some folds end up with few or zero data-points for an outcome so that convergence becomes poor or impossible (which is exacerbated by some biomarkers having few readings). Conversely, stratified cross-validation guarantees that the outcome occurs in the same proportion in each fold and in the same proportion as in the overall training data distribution for that biomarker. To achieve this, per biomarker, patients with and without the outcome were separated and then these groups were shuffled and split into 5 equal subgroups. These groups can then paired at random, ensure training and test datasets have the same proportion of patients with a severe outcome as in full the sample for that biomarker. This substantively improves the chance of convergence for biomarkers with high data missingness.

Here, only complete cases of training data available for each biomarker were considered, i.e. we did not include data for variables marked ‘Test not taken’, to focus on the predictive power of observed test results. The prediction power of the underlying test can be confounded by the clinical decision to order the test only in certain patient circumstances. However, in some cases ‘Test Not Taken’ is much more common (and as described in the results motivating exclusion of the biomarker) and would not represent the predictive power of the biomarker result. In proceeding sections, we allow for a ‘Test not taken’ category, effectively removing the complete cases requirement. Each individual biomarker model includes age and gender (except univariate age and gender models) and were compared against a standard model including only age and gender. Regressions were fit using all associated dummy variables for a given biomarker (e.g. ‘Mild’, ‘Moderate’, ‘Severe’) using ‘Normal’ as the reference.

### Analysis using all valid biomarker data

After individual biomarker evaluation, logistic regression models considering all valid biomarkers (Prediction using individual variables section) and demographic variables were fit to the data. Their predictions were tested via internal and external validation using the stratified cross-validation procedures detailed above, except models were fit using all available training data using ‘Test Not Taken’ for absent data. The models include a standard logistic regression, a logistic regression regularised with LASSO, and two Bayesian models using a flat and a horseshoe prior [16].

### Analysis using reduced variable models

While a model using all biomarker data may have strong predictive power, it is clinically desirable to have a strong prediction with the least amount of biomarkers possible to save on time, money and other resources devoted to biomarker collection and analysis [17, 18]. We used two methodologies to choose reduced variable models to predict COVID-19 severe outcomes, LASSO and Bayesian Projective Prediction.

LASSO is an optimization constraint that shrinks parameters according to their unexplained variance with respect to the outcome variable, reduces over-fitting, and enables variable selection [1]. The optimal degree of regularisation is determined by tuning parameter $\lambda$ within each cross-validation fold through a nested cross-validation step. LASSO has a drawback of having biased coefficient and log-odds estimates, as such after evaluating LASSO models there is a need to run a standard logistic regression model on the reduced biomarker panel selected with the LASSO in order to reduce bias in reporting risk factor effect sizes.

To evaluate LASSO coefficient estimates, we performed repeated nested stratified cross-validation (5-folds for the inner LASSO loop; 5-folds for the outer loop, and 20 repeats). For a particular dataset fit, LASSO optimises for a sparse representation with many coefficients set to zero. Across cross-validated trials these variables will vary. LASSO fits are statistically biased and are better suited as a guide for variable selection, with a reduced variable standard logistic regression used to infer odds ratios. As recommended in Heinze et al. [19], we consider the frequency of how often a particular biomarker has non-zero log-odds coefficients and count across cross-validation trials. There is no set rule for how to translate these frequencies into a set of reduced variables. We suggest to only consider variables that have non-zero coefficients at least 50% of the time, but this is merely a heuristic.

For determining unbiased effect sizes for the reduced variable set with a standard GLM, it was decided that if at least one categorical level for a particular biomarker (e.g. ‘Severe’) was selected by the LASSO, all levels for that biomarker were included in the model. This resulted in a final set of ‘LASSO inspired’ variables that were then fit with standard logistic GLM. Note this approach, and more generally fitting multiple models to the same dataset, is subject to the problem of selective inference (aka multiple comparison error), see [20, 21] and the related R package [22]. This is a limitation that is improved by the Bayesian approach described below.

The second variable selection method explored was Bayesian Projective Prediction [2], a technique for assessing reduced variable models against a complete ‘reference’ model, which in our case is a Bayesian logistic regression with a horseshoe prior [16]. Priors such as the horseshoe can be applied to provide adaptive shrinkage to covariates in Bayesian models directly so that full posterior distributions of odds estimates can be generated in an unbiased way. Unlike the LASSO, this does not shrink coefficients to zero exactly as the inherent uncertainty is not ignored. To perform hard variable selection, the recent approach of Projective Prediction can be used to compare the fit of sub-models of the reference model through projections and approximate leave-one-out (LOO) cross-validation. Under the hood, Projective Prediction uses forward search to select submodels for comparison, but retains the Bayesian inference for coefficient ranking and odds-ratio estimates. The projective prediction package allows systematic evaluation of the trade-off between AUC performance and the ranked contribution from the variables included in the model. The experimenter must decide at what performance level to cut-off the ranked variables. We chose to examine when increases in AUC asymptoted and used any biomarkers that did not have ‘Test Not Taken’ as the highest ranked predictor. This reduced set of variables was used for the submodel projection. Projective prediction allows the flexibility to train one model on all valid available data, perform variable selection, and then use any projected sub-model with reduced variables to predict outcomes for novel data. Projective prediction models were evaluated using cross-validation procedures described in prior sections. Note, the analysis of the projective prediction model using all training data uses LOO for variable selection, which is computationally intensive. To speed variable selection computation during our cross-validation analysis, we used ‘naive’ variable selection, which only considers the training data from current fold as is, and does not perform any further internal cross-validation (the projective prediction package allows naive, k-fold, and LOO).

## Results

### Cohort description

756 of 1159 patients (73%) patients testing positive for SARS-CoV-2 were eligible given our inclusion criteria, see Fig. 1. Of these patients, 57% were hospitalised for COVID-19 (n=433), the remainder (n=323) had nosocomial infection. For our statistical models, the training cohort (n=534) was defined as all adults admitted to hospital and testing positive for SARS-Cov-2 by PCR, or testing positive while already admitted between March 1 to October 31, 2020. For external validation, we held the DGH cohort (n=222) out of training. This cohort was selected as the hospital is in another county compared to the trusts used in the training data. To avoid over-fitting to local idiosyncrasies, ideally, the external validation data set would differ on a national or international level. Given our limited data, this was the best external validation possible. Patients in the training set had a mean age of 70, were 44% female, and 28% had severe outcomes. The external validation set had a mean age of 74, were 47% female, and 37% had a severe outcome. There were statistically significant differences (tested via Wilcoxon Mann-Whitney U test evaluated at a significance level of 0.05), with the external validation set having a larger incidence of severe outcomes (W = 64296, *p*-value = 0.02), and an older population (W = 68074, *p*-value < 0.001). Gender was statistically similar (W = 57480, *p*-value = 0.44)

### Prediction using individual variables

Table 2 shows descriptive statistics on individual biomarker readings and their odds ratio contributions in a 5-fold 20-repeat stratified cross-validated logistic regression including the particular biomarker and age and gender. Our approach uses complete cases to estimate the predictive capacity of biomarker test results (avoiding ‘Test Not Taken’) but this may introduce bias as discussed. Table 3 details performance using the area under the receiver operating characteristic curve (AUC) metric, comparing biomarker models (a particular biomarker plus age and gender) to a model using only age and gender. A simple age and gender model acts as a foil to illustrate the worth of a biomarker over easily collected but often predictive variables. Due to the categorical representation of the biomarkers, individual levels may be significant while another is not (e.g. ‘Severe’ is a predictor, but ‘Mild’ is not). Statistically significant predictors (i.e. odds ratios deviating from one with *p*-value at 0.05 or lower) associated with increasing risk of a severe outcome (as shown in Table 2) include age, and the biomarkers: Activated Partial Thromboplastin Time (Mild), Prothrombin time (Abnormal), blood pH (Abnormal), Haemoglobin (Severe), Platelet count (Moderate), Lymphocytes (Moderate, Severe), Neutrophils (Severe), Neutrophil-Lymphocyte Ratio (Mild, Moderate, Severe), C-Reactive Protein (Abnormal), Urea (Abnormal), and Troponin-T (Abnormal). Nosocomial transmission was included due to the high number of cases in our cohort but was not a significant predictor and excluded from further analyses. Due to small numbers preventing cross-validation, Triglycerides, Glycated Haemoglobin, Procalcitonin (also invalid due to being recorded only in ICU), Fibrinogen, and Lactose Dehydrogenase were excluded from further analysis and require future research.

**Table 2:**
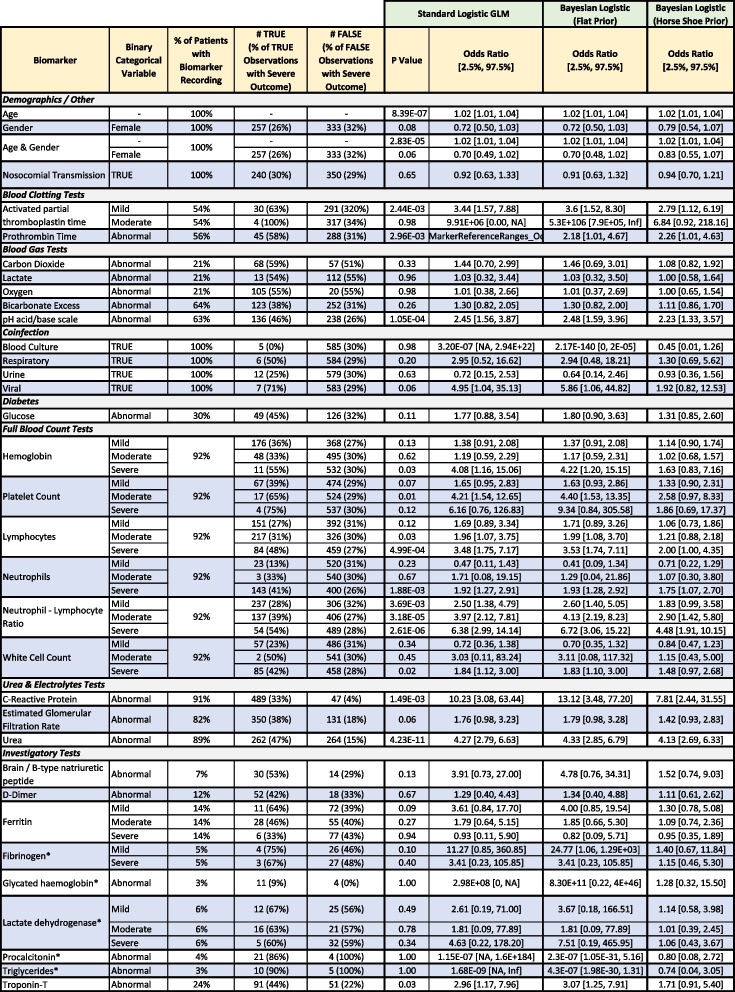
Individual biomarker evaluation including descriptive statistics, unadjusted *p*-values, and logistic regression model outcomes (Standard, Bayesian with flat prior, and Bayes with horseshoe prior), including age and gender (except univariate age and gender models)

^*^Biomarkers not included in subsequent models due to small sample size, and recorded only in ICU (PCT)

The True and False columns describe the number/percentage of severe outcomes for cases where the particular biomarker or demographic reading is true or false. For example, there were 257 patients who were women who had a severe outcome, and conversely there were 333 patients who were not women (i.e. men) who had a severe outcome. Regressions were fit using all associated dummy variables for a given biomarker (e.g. normal, mild, moderate, severe) and using only complete cases of training data, i.e. not using a variable for ‘Test not taken’. Categorical variables use a reading of ‘Normal’ as a reference in the fitted model, except ‘Male’ used as the reference category for gender

**Table 3:**
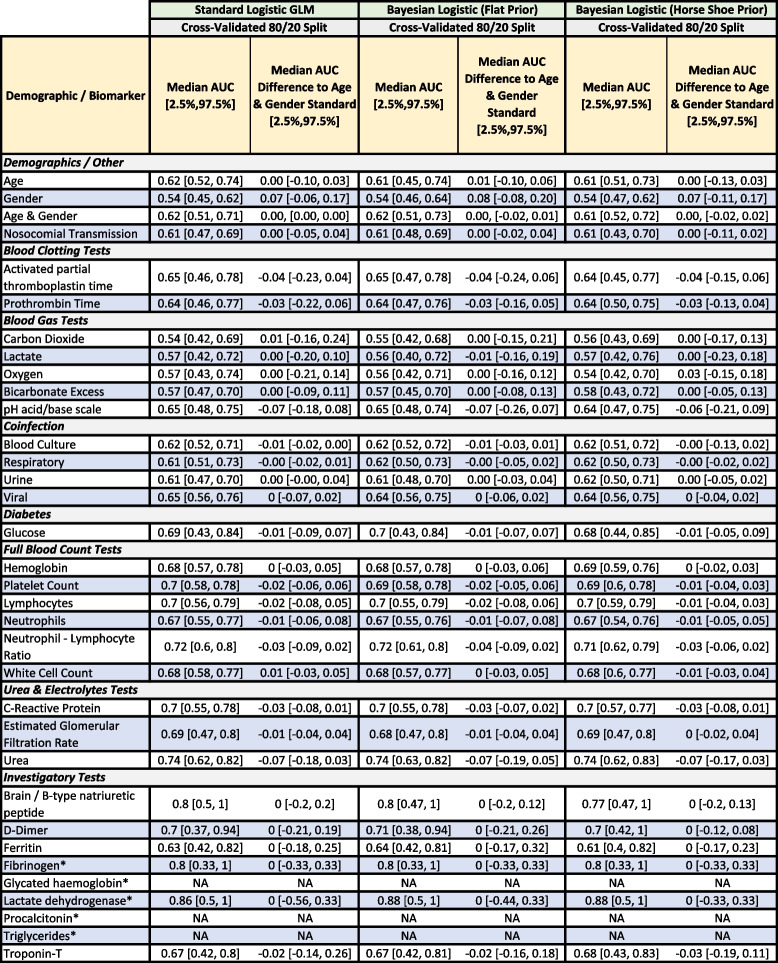
Predictive performance of the individual biomarker models in Table 2 as described by the median area under the curve (AUC) in receiver operating curve (ROC) analysis and median difference between an Age and Gender reference model and the same model (negative values indicate the reference has worse performance) with the particular biomarker included (except univariate age and gender models)

^*^Biomarkers not included in subsequent models due to small sample size, and recorded only in ICU (PCT)

Regressions were fit using all associated dummy variables for a given biomarker (e.g. mild, moderate, severe) and using only complete cases of training data (n=590), i.e. not using a variable for ‘Test not taken’. 95% inter-quantile ranges calculated via 5-fold cross-validation with 20 repeats (100 models total). Categorical variables use a reading of ‘Normal’ as a reference in the fitted model, except ‘Male’ used as the reference category for gender

### Regression models using all valid biomarker data

Each model was evaluated via 5-fold stratified cross-validation with 20 repeats (100 models total). As such, each model is trained with a randomised sample of 80% of the training data set (n=427). Internal validation evaluates model predictions on the 20% (n=107) held out. External validation uses the same model, but is instead tested on the never trained on external validation data set on recorded a separate hospital (n=222). Missing data for each biomarker is coded as ‘Test Not Taken’ and is included as a predictor variable. Table 4 shows the performance of these models (AUC, Sensitivity, Specificity).

To estimate variability in model performance and allow comparison between models, we compute inter-quantile AUC difference ranges using 5-fold 20-repeat cross-validation of models. While Delong’s method [23] is also be used to compare between models, it tests only for a significant difference between the AUCs of two trained models. Conversely, cross-validation (or bootstrap) considers also variability in model training due to sample variance by providing a comparison across models for each of many data splits. For each data split, we compute the AUC for a given model and then compute the delta to the reference model (Bayesian horseshoe), thus allowing the comparison of 95% intervals. Cross-validation results provide 95% inter-quantile ranges that clearly illustrate that in general, all models perform similarly, with a median AUC ranging from 0.76–0.82 in internal validation, and ranging from 0.67–0.71 in external validation. While the LASSO inspired GLM model has the best median internal AUC difference (0.02 better than the Bayesian horseshoe reference), all models overlap in their 95% AUC difference intervals. When considering external validation, the median AUC difference tends to be smallest or even slightly positive for the Bayesian methods, but all models overlap within the 95% bounds of the reference model, except the LASSO model. Note the LASSO model also has higher variation in AUC difference indicating the model’s performance is not very consistent across cross-validation folds. The calibration of the models is varied on the internal training data, with the GLM with LASSO regularization and Bayesian and projective prediction models having the best performance. However, the flat and horseshoe Bayesian models appear to overestimate the presence of severe events as indicated by the calibration-in-the-large values. External validation calibration is worse across models with most underestimating the presence of severe events. While the 4-biomarker projective prediction model has good AUC performance the external calibration slope is quite low (0.19) which appears to be due to poor estimates for patients with high probabilities of a severe outcome, see Supplementary Materials A9.

**Table 4:**
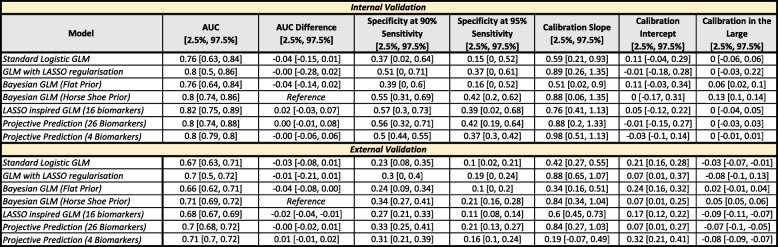
Internal and external cross-validated performance of models trained using valid biomarker data

95% inter-quantile ranges are presented for each estimate. Specificity is obtained by evaluating at a set sensitivity of either 90% or 95%. All reduced variable models include age, and a stated number of biomarkers. The reduced variable standard GLM uses age and 16 biomarkers that had non-zero coefficients on $>=$50% LASSO Cross-validation trials. If at least one categorical level for a particular biomarker (e.g. severe) met this requirement, all levels for that biomarker were included in the model. The 4 biomarker projective prediction model uses all categorical levels for Urea, PT, CRP, and NLR. Pairwise AUC difference is presented in comparison to the Bayesian (Horse shoe prior) model

### Reduced variable models

The models detailed above are moderately good predictors of severe COVID-19 outcomes, but for clinicians with limited time and resources, reduced models can balance predictive performance with ease of clinical use by using only the most informative biomarkers. To address this, we use two variable selection approaches, LASSO and projective prediction, that allow the creation of reduced models with fewer biomarkers but similar performance to the larger models.

### LASSO models

After performing 5-fold 20 repeat cross-validation we examined the frequency of how often a particular biomarker has a coefficient greater than zero and count across cross-validation trials. Supplementary Figure A10 shows the frequency of variables having a coefficient great than zero in the cross-validated LASSO analysis. If we select variables that appear at least 50% of the time, our reduced model would include: Age, BE (abnormal), CRP (abnormal), eGFT (abnormal), HB (severe), PLT (mild, moderate), Lymphocytes (Severe), Neutrophils (Mild, Severe), NLR (Severe), APTT (mild, moderate), 0xygen (abnormal), PT (abnormal), blood pH (abnormal), Urea (abnormal), and positive viral, respiratory, and blood culture co-infections.

For the LASSO inspired reduced variable standard GLM, this resulted in a model using the 16 biomarkers above and age for all categorical levels, and was evaluated via both cross-validation and as fit to all available training data. This model had performance similar to the models using all valid biomarker data, with a median external validation AUC of 0.68 [0.67, 0.69], see Table 4.

Note, ‘Test Not Taken’ was a significant predictor for some biomarkers on over 50% of cross-validation trials (see Supplementary Figure A10). The potential significance of missing data is complex and is addressed in the Discussion section. Due to this confounding, biomarkers whose top predictive contribution was from ‘Test Not Taken’ were excluded from both LASSO reduced variable models and projective prediction models described below.

### Projective prediction models

When all biomarkers are considered, projective prediction ranks all variables in descending order of contribution to AUC performance. We considered the top 20 including: Urea (abnormal), Age, PT (abnormal), CRP (abnormal), NLR (Severe), APPT (moderate), PLT (mild, moderate), Neutrophils (mild, severe), Lymphocytes (severe), blood co-infection, hemoglobin (severe), blood pH (abnormal). Thus age and 11 biomarkers were candidates for a reduced model. Several predictors of ‘Test Not Taken’ were in the AUC ranking. However, as mentioned above, these biomarkers are set aside due to this confound. Supplementary Figure A11 shows the projective prediction ranking the AUC contribution. A model using a projection incorporating all biomarker and demographic data is equivalent to the standard Bayesian GLM we evaluated in the prior section, see Table 4.

Reduced variable projections were evaluated by manual inspection of AUC performance among groups of models using the top biomarkers. Guided by the projective prediction ranking, we ran a model using only the top biomarker, using only the top two, the top three, and so on. As described above, we omit biomarkers with significant contributions from ‘Test Not Taken’ and include all categorical levels for a given biomarker as long as one level is highly ranked. Ultimately, we found a four biomarker projective prediction model using age and including urea, prothrombin time, neutrophil-lymphocyte ratios, and C-reactive protein had similar performance to larger models with a median internal validation AUC of 0.8 [0.79, 0.8], and external validation AUC of 0.71 [0.7, 0.72], as shown in Table 4. Odds ratios for the full Bayesian model and the reduced 4-biomarker model can be found in Supplementary Materials A12.

The 11 coefficients and intercept present can be substituted into a standard logistic equation. The calibration of the model is reasonably good on the training data but has poor calibration on the external validation set, see Supplementary Figure A13.

## Discussion

### Summary

Building prediction models using real world clinical data offers many challenges. There are numerous decision points required to curate data and many choices that require domain expertise. We use a COVID-19 dataset with novel biomarker data to illustrate many of these challenges. Furthermore, if models are to be used clinically they must be feasible given the many resource constraints clinicians face. In principle, a model like ours (with a larger training set, testing, and translation into a clinical score) could have been used to guide clinicians on how to triage patients and direct prophylactic measures (though these were minimal at the time) and help anticipate which patients would be more at risk. While our model is not of use at this stage with COVID-19, our methodology would generalise to other infectious diseases.

We demonstrate methods for Bayesian variable selection in logistic regression using projective prediction and compare to a LASSO approach. While Bayesian models and projective prediction are more computationally intensive than standard approaches, they offer small but consistent AUC gains. A Bayesian approach also provides unbiased coefficients compared to LASSO, and projective prediction provides a systematic method to evaluate the contribution of model variables by AUC contribution and guide variable selection. Below we detail many of the methodological challenges faced.

### Challenges of complex medical data

Data curation is challenging as clinical data are heterogeneous in multiple ways. Biomarkers are recorded for different reasons, e.g. routine upon admission, investigatory tests, or tests primarily or exclusively taken in ICU. Further, some biomarkers are typically recorded together (but not always) as part of a test suite, including: Urea and electrolytes, full blood count, COVID-19 and co-infection swab test, blood clotting, and blood gas tests (arterial or venous). The schedule when these markers are recorded varies by patient and clinical decision, leading to records being present in highly varying amounts, e.g. only 3% up to 100% of patients depending on the particular biomarker, see Supplementary Materials A1.

### Modelling choices

When constructing and evaluating models, there are many choice points that should be explicitly highlighted with justification, be it based on convenience, computational complexity, clinical advice, or a heuristic. We regularly consulted our clinical partners for choices on the transformation of variables, the time window to consider, why data was missing and patient inclusion criteria. Complex data sets can be modelled with a variety of approaches, as described below we considered a number of time windows, ways to aggregate multi-day data, and data imputation procedures before forming a consensus with the presented models. Our approach emphasizes explainable risk factors, predictive performance and highlights the benefits of the Bayesian variable selection technique projective prediction for practical clinical use. However, non-linear approaches such as decision forests, boosting, or neural networks are all valid options if these features are not prioritized.

### Missing data

Missingness, in the context of this study and in healthcare data more generally, can sometimes be informative and missing not at random, with the presence or absence of a test correlated with the its measurement or the study outcome. Imputation of missing data relies on key statistical assumptions that imputed variables are missing at random (MAR) or missing completely at random. Conversations with our clinical co-authors established some routinely collected biomarkers might be inferred to be MAR. However, the routines identified were specific to a small a subset of our cohort and not likely to extrapolate. Clinicians advised that tests not being taken are almost always a clinical decision and therefor not random, as such we ultimately erred to be conservative and avoid all imputation, and instead include the presence/absence of missing values as a covariate itself [24, 25]. As such, in the current study we chose to use placeholders for ‘Test not taken’ if there was no recorded value for a particular biomarker within the evaluated 3-day window after the key date.

This approach allows the possibility that a ‘Test Not Taken’ may be a significant predictor. This has many potential meanings, as it may convey that when a patient is doing well and unlikely to experience a severe outcome, clinicians are unlikely to request some biomarker tests. Alternatively, if a patient is in palliative care and has a poor prognosis, a clinician may consider further testing unnecessary. As such, the likelihood of a test being administered may follow an inverted-U function as patients to healthy or too ill may not have tests administered. Furthermore, as our data was collected early in the pandemic, there may be other underlying clinical decisions or resource limitations that drove why some tests were taken but not others. Lastly, because we only consider results from within the first 3 days after a patients date, it may be that some tests were simply taken later in a patient’s stay due to operational constraints, and hence may be more predictive as they were taken closer to the outcome. When these instances occurred, we were conservative and excluded biomarkers with ‘Test Not Taken’ as the most informative category from our reduced variable models.

### Data transforms - time windows

In the early days of the COVID-19 pandemic clinicians desired a way to triage patients near admission to help manage resources. If a good prediction on patient outcome could be made on or near the time of admission, this could greatly help divert resources to the correct patients. However, not all tests are administered on admission. To balance inclusion of test data not available on the day of admission and the need for clinical decisions to be guided soon after admission, we chose to consider the first value recorded for each biomarkers within three days after their ‘key date’, i.e. date of admission if already COVID-19 positive, or if already in hospital, the date of testing COVID-19 positive. However, given the richness of the time series data collected, further research into models that leverage this extra information is needed.

Focusing on early detection reflects the intent for the model to improve early stage clinical decision making when potential treatments or changes in care may be introduced. This focus on the first reading in a 3-day interval loses information, but greatly simplifies the modelling approach. Note, this choice is not without risk of reducing statistical power, increasing the risk of false positives, and underestimation of the extent of variation in biomarker readings and outcomes between groups [26]. It is likely that representing biomarker data as time-series (assuming regular measures across patients) would add considerable information for continuous monitoring.

### Data transforms - continuous vs. categorical

A key modelling decision must be made on whether to use continuous data or transformed categorical data. Clinicians often use biomarker thresholds to provide semantic categories (e.g. normal, mild, moderate, severe) which sometimes use non-linear or discontinuous mappings that require special care if using continuous data. While clinical thresholds are likely established with evidence, it may be the case that thresholds for one use may not apply to a novel use. This led [12, 27] to use machine learning approaches to build categorisation models on continuous biomarker data dependent on the training data at hand. However, using machine learning to establish categorisation thresholds on our biomarker data is difficult with a small training data set and the heterogeneity of biomarker recordings. If missing data imputation is performed, it raises another decision point on whether to impute the continuous or the transformed categorical data.

Another important factor to recognise is that some biomarkers lack a linear relationship between a reading and a semantic category. Biomarkers can have a lower and upper bound for what is considered normal, and both below and above this range reflects clinically meaningful yet sometimes separate abnormalities. The modelling needs to factor in non-linearity when persevering continuous data or trying to map to a categorical space. In our position, categorical transformation had an advantage, as they allowed us to collaborate with ICU consultants while using pre-established clinically acceptable ranges to define our categorisation, see Table 1. Categorization is worth critical consideration in model planning and potentially worth revisiting. For example, with eGFR we simply consider kidney function as normal or abnormal, but test results can be put into more fine-grained categories to label the severity of kidney failure.

### Training and external validation data selection

There are multiple ways that our data set could be split between training and external validation sets, e.g. randomly sampling 1/3 of the data to hold out as an external validation set. Random selection of training data should in principle generate data more representative of the external validation set left out. However, hospitals may have differing practices and non-stratified randomization may inflate performance at the cost of real world generalisation. We chose to separate our training and external validation datasets by hospital to provide a stronger test of generalisation that should mimic generalisation to novel hospitals completely outside the original training data .

### Model performance evaluation and dissemination

There are a variety of ways statistical model performance can be evaluated. Here we have chose here to emphasize cross-validated estimates of AUC, sensitivity, and specificity. Inter-quartile intervals over these measures reveal that the variety of models perform in similar ways. With a larger data set, trade-offs may become more apparent. Model calibration on the external validation set is a clear weak point. While the models have a reasonable calibration for training data, generalization performance is weak and suggestive of the lack of sufficient data.

### Comparison to contemporary models

We found several biomarkers previously highlighted by other groups to have significant predictive power, including: Urea, Neutrophil-Lymphocyte Ratio (NLR), Lymphoctyes, PT, eGFR, and CRP. Our highly reduced 3-biomarker model (plus age) uses Urea (highlighted by all prior models), NLR (highlighted by [13, 27, 28]), and PT (highlighted by [27, 29]). These biomarkers highlight aspects of hypovolaemia (UREA), inflammation (NLR and CRP), and blood clotting factors (PT) that are consistently altered in patients with severe outcomes. A direct comparison with other models is not possible due to differing variables, but our external validation performance (Full model AUC: 0.7, 3-biomarker model AUC: 0.67) suffers compared to Knight et al (AUC: 0.77) and is similar to Carr et al (AUC: 0.69 to 0.79 dependent on the training dataset). While our current model is not state of the art, with a larger more diverse dataset, our methods should achieve such results and allow possible inclusion of some biomarkers not included in the present model, as well physiological bedside measures captured by Knight et al. and Carr et al. but not present in our own.

### Advantages of Bayesian modelling

While the predictive performance across models presented here is generally similar within 95% bounds, the Bayesian horseshoe model has slightly better median AUC difference cross-validated predictive performance. Reasons for researchers to favor Bayesian approaches should include that coefficients estimated via Bayes should on average deliver better predictive performance than standard GLM [30]. Additionally, if a sparse model is needed, a horseshoe prior can provide advantages similar to LASSO without biased coefficient estimates enabling joint probabilistic modelling of prediction and risk factor inference. Computationally, Bayesian techniques can be slow due the Hamiltonian Monte Carlo used to sample the coefficient space. If one is interested in variable selection, projective prediction offers the ability to take a single Bayesian model fit, run a variable selection algorithm to rank variable contributions, and then arbitrarily create sub-model projections with any number of original variables. While the initial model fit and variable selection are computationally intensive, sub-model projections are fast to create and performance test. Bayesian logistic regression with variable selection has the flexibility of providing both conventional risk factor analysis and prediction, but approaches like deep learning [31] or ensemble methods [32] can offer superior prediction performance due to their non-linear nature. However, there are trade-offs, deep learning works best with large datasets (unlike ours) and does not have intuitive regression coefficients for explainability. Ensemble methods (e.g., gradient-boosted decision trees) can achieve high performance with smaller datasets but also with some sacrifice in explainability. Further, neither of these approaches have a statistically rigorous variable selection method similar to projective prediction, though models can be augmented with regularization terms to encourage sparsity. However, tools like SHAP [33] are becoming more mature and can offer a model agnostic way to view contribution of both variables and samples to model performance, and is well worth exploring. Ultimately, we favored the clinical explainability offered by logistic regression and ease of use with the ProjPred package for variable selection, even if it does sacrifice some performance compared to non-linear techniques. We encourage researchers to try a variety of models depending on requirements for balancing data set size, performance, and explainability.

## Conclusion

*Limitations:* This is a retrospective cohort study in Southwest England where case numbers have varied widely, and were below national incidence rates during the first wave. This results in less precise parameter estimates for prediction models (less power/smaller sample size) and likely reduced generalizability of the model to other settings. The timing of biomarker collection was highly varied both within and between patients, with many types of readings missing.

*Strengths:* The primary strength of our study is the granularity of serial laboratory data available linked to clinical outcomes. This study was performed during the first wave where there was the original Wuhan strain circulating amongst the unvaccinated naïve population without any specific immunomodulating therapies such as steroids or antiviral agents, reflecting the “true” homeostasis derangements at a population level.

In particular, this study describes the variety of challenges present in complex medical data sets and how researchers need to balance the aim of statistically sound practices with the pragmatics and limitations of observational datasets like these. We highlight the benefits of recent Bayesian methodology for variable selection. Our study reiterates the predictive value of previously identified biomarkers for COVID-19 severity assessment (e.g. age, urea, prothrombin time, c-Reactive protein, and neutrophil-lymphocyte ratio). Both the full and reduced variable models have moderately good training performance, but improved external validation is needed for all models to be clinically viable. The methods presented here should generalize well to a larger dataset and serve as a guide.

## Supplementary Information

Supplementary Material 1.

## Data Availability

Due to strict data governance of medical records, the data in this study cannot be directly shared by the authors. Interested parties should contact the corresponding authors and bnssg.research@nhs.net to arrange a data sharing discussion with the Bristol, North Somerset, and South Gloucestershire Integrated Care Board who steward the data, for more information please consult the website: BNSSG Research and Evidence.
