# Supplementary material for: Comparing conventional and Bayesian workflows for clinical outcome prediction modelling with an exemplar cohort study of severe COVID-19 infection incorporating clinical biomarker test results
Source: BMC Med Inform Decis Mak. 2025 Mar 10;25:123. doi: 10.1186/s12911-025-02955-3 (PMC11892292; doi:10.1186/s12911-025-02955-3)
Supplement: Supplementary file 1 — Supplementary Material 1. [file 12911_2025_2955_MOESM1_ESM.pdf]

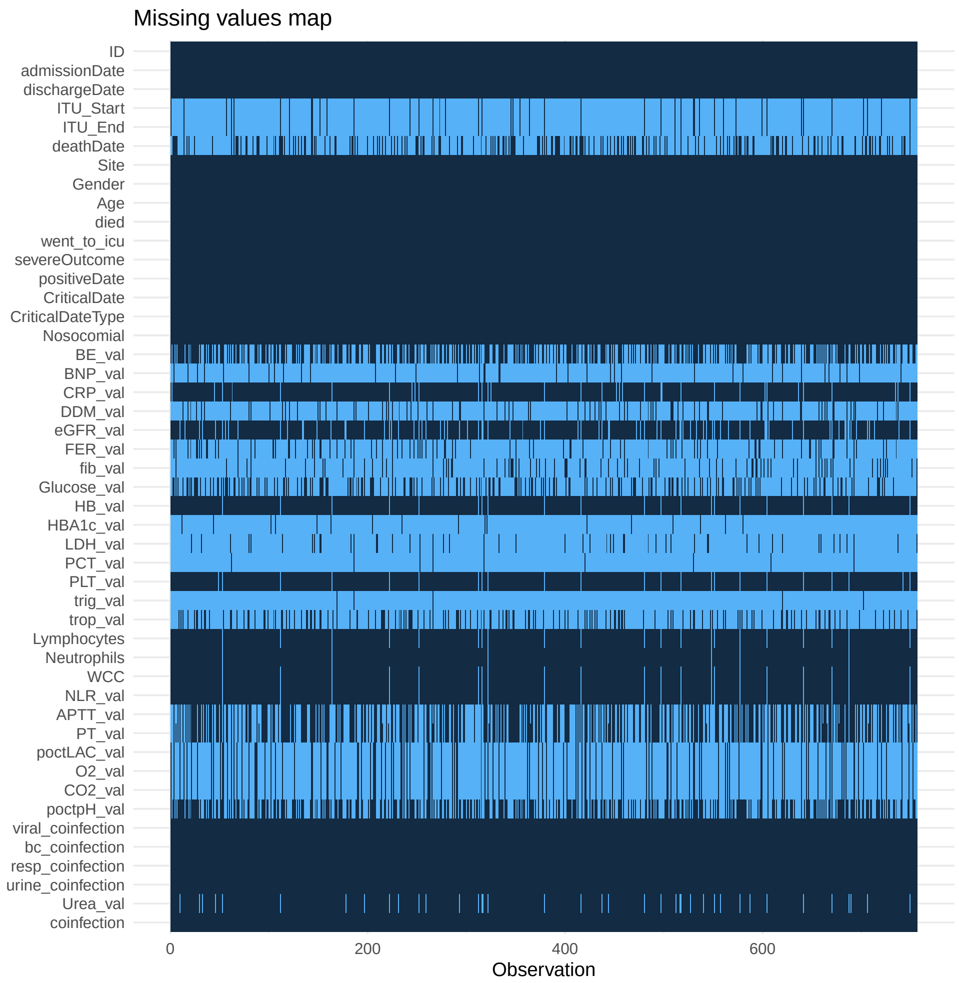

Fig. A1: Heat map displaying missing values across recorded biomarkers. Light blue indicates a value is missing and dark blue indicate it is present

Appendix A Appendix

The appendix figures expand on the challenges presented with patient level data and elaborate on regression model performance.

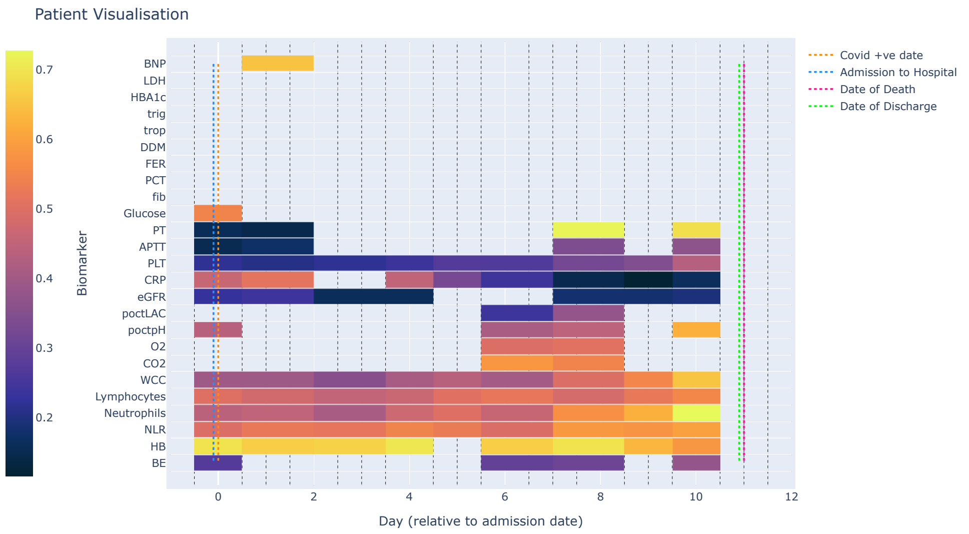

Fig. A2: Example biomarker time series for a patient admitted to hospital COVID-19 positive and who subsequently died almost two weeks later.

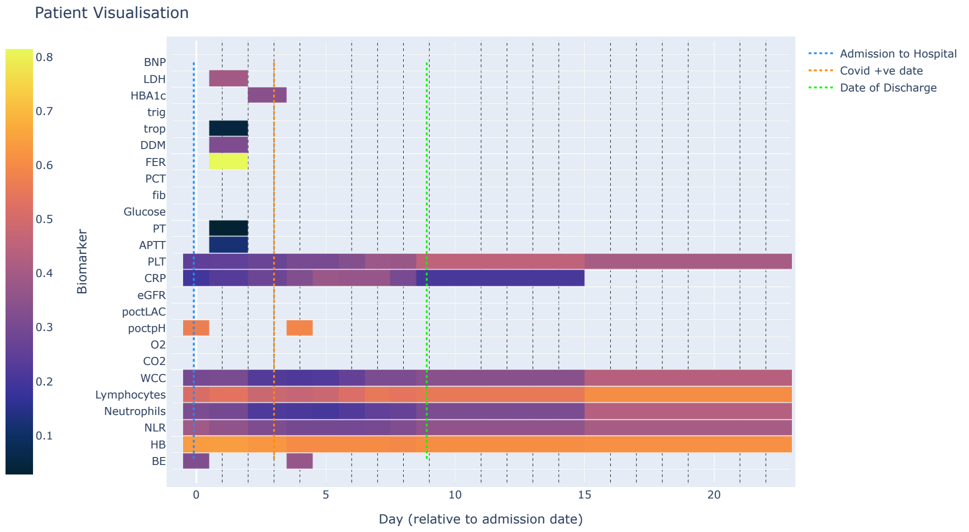

Fig. A3: Example biomarker time series for a patient admitted to hospital with subsequent nosocomial transmission and discharge a week later.

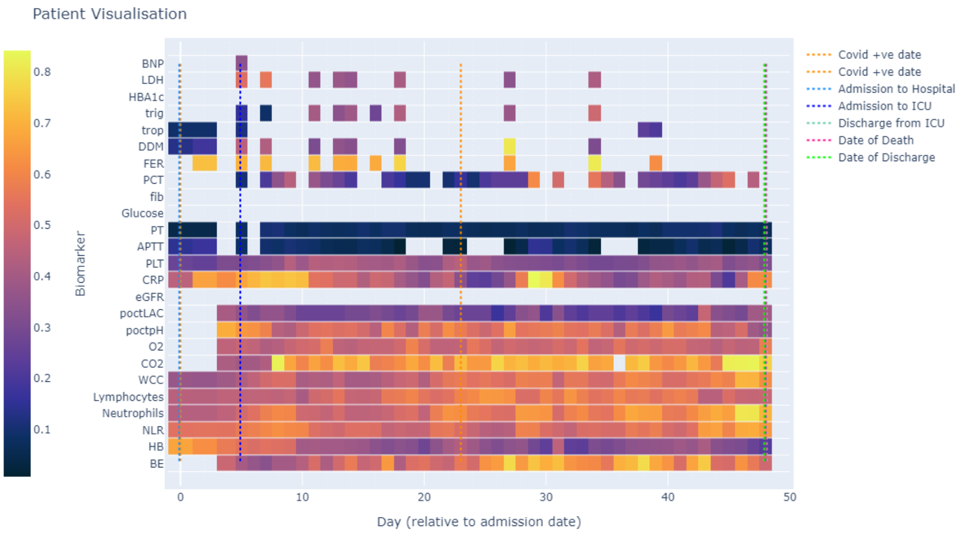

Fig. A4: Example biomarker time series for a patient admitted to hospital COVID-19 positive, with subsequent entrance to ICU and death over one month later.

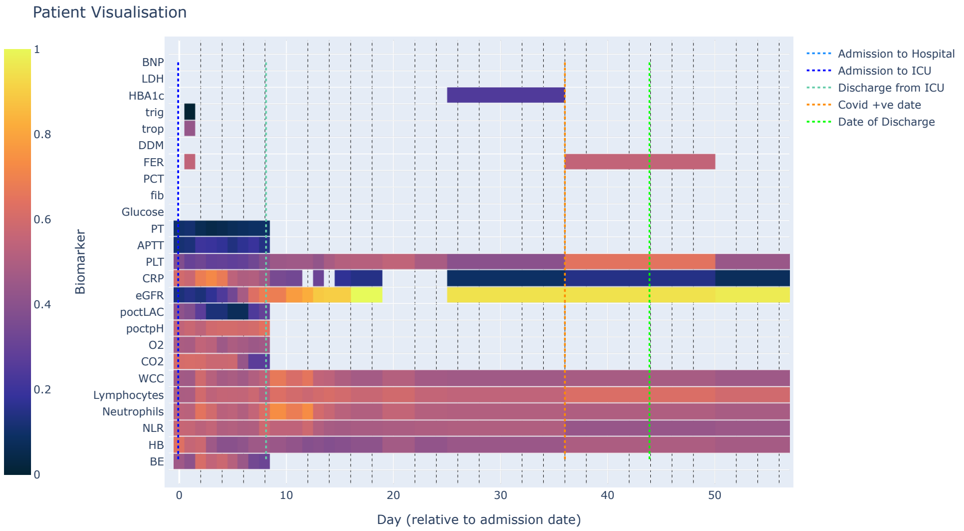

Fig. A5: Example biomarker time series for a patient admitted to hospital and ICU, with subsequent nosocomial transmission and discharge about one week later.

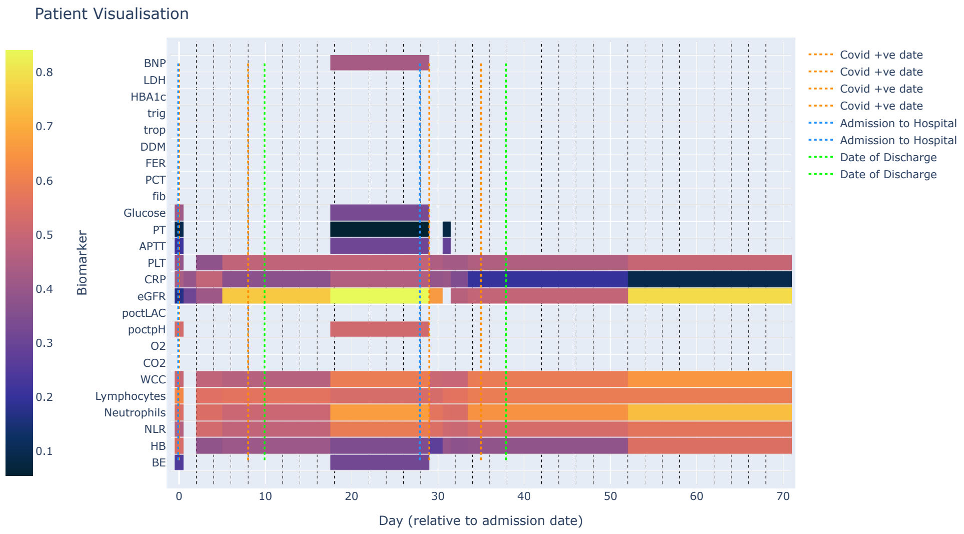

Fig. A6: Example biomarker time series for a patient with two hospital admissions and testing COVID-19 positive on the first, with discharge almost two weeks after second admission.

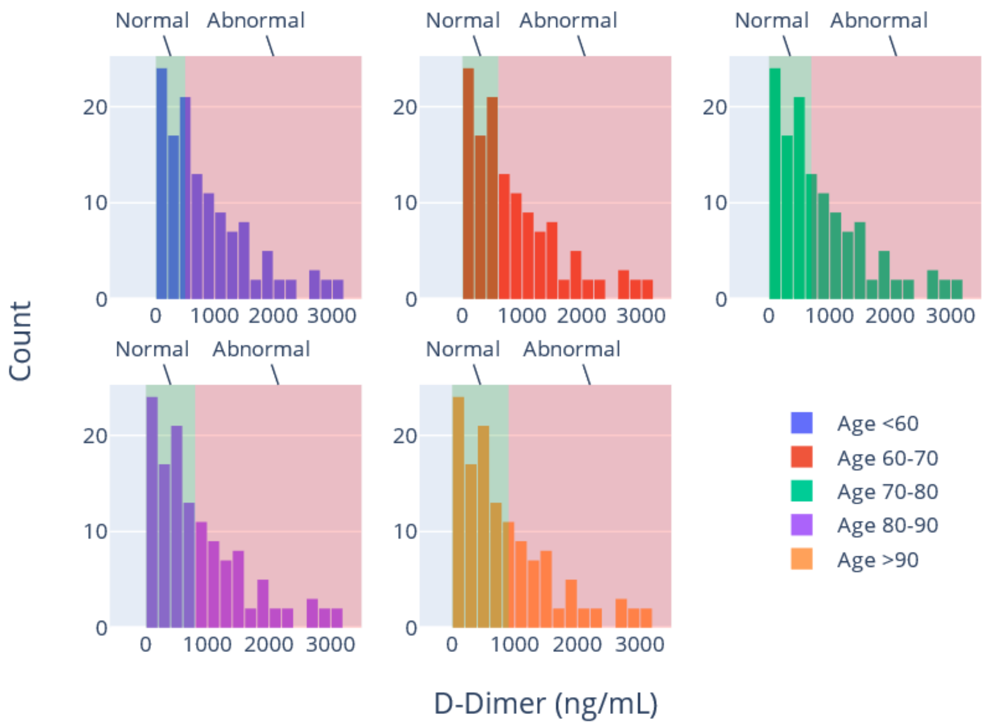

Fig. A7: Distribution of D-Dimer readings with clinical classification requiring age and gender bands

Biomarker	Binary Categorical Variable	P-Value	Odds Ratio CI [2.5%, 97.5%]
Demographics / Other			
Age	-	1.46E-04	1.05 [1.02, 1.07]
Gender	Female	0.49	1.2 [0.71, 2.04]
Blood Clotting Tests			
Activated partial thromboplastin time	Mild	0.33	1.82 [0.55, 6.03]
	Moderate	0.99	4.9e+07 [0, Inf]
	Not Taken	0.83	0.81 [0.12, 5.43]
Prothrombin Time	Abnormal	0.01	3.76 [1.29, 10.91]
	Not Taken	0.72	1.42 [0.21, 9.86]
Blood Gas Tests			
Carbon Dioxide	Abnormal	0.74	0.83 [0.29, 2.42]
	Not Taken	NA	NA
Lactate	Abnormal	0.64	0.69 [0.14, 3.4]
	Not Taken	0.16	0.28 [0.05, 1.64]
Oxygen	Abnormal	0.48	1.79 [0.36, 8.93]
	Not Taken	NA	NA
Bicarbonate Excess	Abnormal	0.14	1.66 [0.85, 3.24]
	Not Taken	NA	NA
pH acid/base scale	Abnormal	0.56	1.22 [0.64, 2.33]
	Not Taken	1.00	1.1e+07 [0, Inf]
Coinfection			
Blood Culture	TRUE	0.99	0 [0, Inf]
Respiratory	TRUE	0.20	4.59 [0.45, 46.35]
Urine	TRUE	0.77	0.75 [0.11, 5.14]
Viral	TRUE	0.07	11.37 [0.86, 150.44]
Diabetes			
Glucose	Abnormal	0.25	1.78 [0.67, 4.74]
	Not Taken	0.64	1.18 [0.59, 2.33]
Full Blood Count Tests			
Hemoglobin	Mild	0.38	1.29 [0.73, 2.3]
	Moderate	0.88	0.93 [0.37, 2.35]
	Severe	0.20	4 [0.48, 32.96]
	Not Taken	0.99	2.5e+18 [0, Inf]
Platelet Count	Mild	0.01	2.86 [1.33, 6.11]
	Moderate	0.03	5.97 [1.25, 28.56]
	Severe	0.82	1.66 [0.02, 131.4]
	Not Taken	0.99	0 [0, Inf]
Lymphocytes	Mild	0.35	1.54 [0.63, 3.79]
	Moderate	0.31	1.69 [0.62, 4.61]
	Severe	0.09	3.07 [0.83, 11.36]
	Not Taken	NA	NA
Neutrophils	Mild	0.04	0.07 [0, 0.87]
	Moderate	0.99	1.05 [0, 329.16]
	Severe	0.38	1.46 [0.62, 3.43]
	Not Taken	0.98	0 [0, Inf]
Neutrophil - Lymphocyte Ratio	Mild	0.74	1.16 [0.48, 2.79]
	Moderate	0.98	1.01 [0.32, 3.22]
	Severe	0.57	1.59 [0.32, 7.9]
	Not Taken	NA	NA
White Cell Count	Mild	0.81	0.88 [0.31, 2.48]
	Moderate	0.97	0.9 [0, 372.32]
	Severe	0.68	1.23 [0.46, 3.33]
	Not Taken	NA	NA
Urea & Electrolytes Tests			
C-Reactive Protein	Abnormal	0.02	15.4 [1.66, 142.81]
	Not Taken	0.59	2.46 [0.1, 62.87]
Estimated Glomerular Filtration Rate	Abnormal	0.65	1.22 [0.51, 2.94]
	Not Taken	0.09	0.3 [0.07, 1.23]
Urea	Abnormal	2.48E-03	2.43 [1.37, 4.33]
	Not Taken	0.74	1.42 [0.18, 11.42]
Investigatory Tests			
Brain / B-type natriuretic peptide	Abnormal	0.26	5.17 [0.29, 92.25]
	Not Taken	0.31	3.92 [0.28, 54.02]
D-Dimer	Abnormal	0.32	0.35 [0.05, 2.72]
	Not Taken	0.57	0.6 [0.1, 3.45]
Ferritin	Mild	0.19	5.05 [0.45, 56.98]
	Moderate	0.34	2.17 [0.45, 10.47]
	Severe	0.74	0.6 [0.03, 11.92]
	Not Taken	0.80	1.15 [0.39, 3.42]
Troponin-T	Abnormal	0.64	0.72 [0.19, 2.79]
	Not Taken	0.58	0.7 [0.2, 2.46]
Intercept			
Intercept	-	0.004	0.001 [0.0, 0.11]

Fig. A8: Standard logistic regression odds ratio and confidence intervals per biomarker using all valid biomarker training data available (n=534). Note most biomarkers include a 'Test Not Taken' stand in variable.

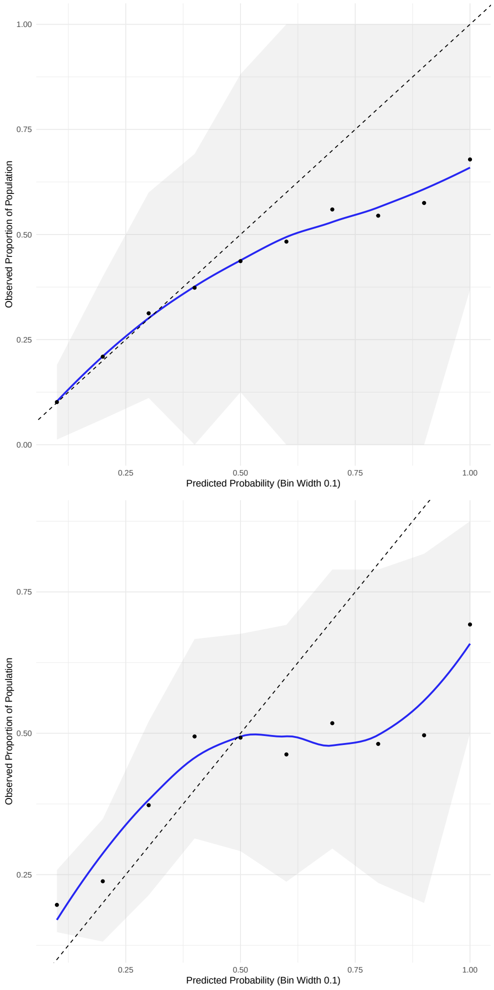

Fig. A9: Model calibration depicting a standard GLM model trained on: (Top) training data (n=534) and tested on the same; (Bottom) training data and tested on external validation data (n=222). The blue line is a loess smoothed curve across all cross-validation trials. Black circles represent the median value across trials. The grey shaded area indicates the 95% observed interval. A well calibrated model should evenly distribute outcome probabilities, i.e. be close to unity.

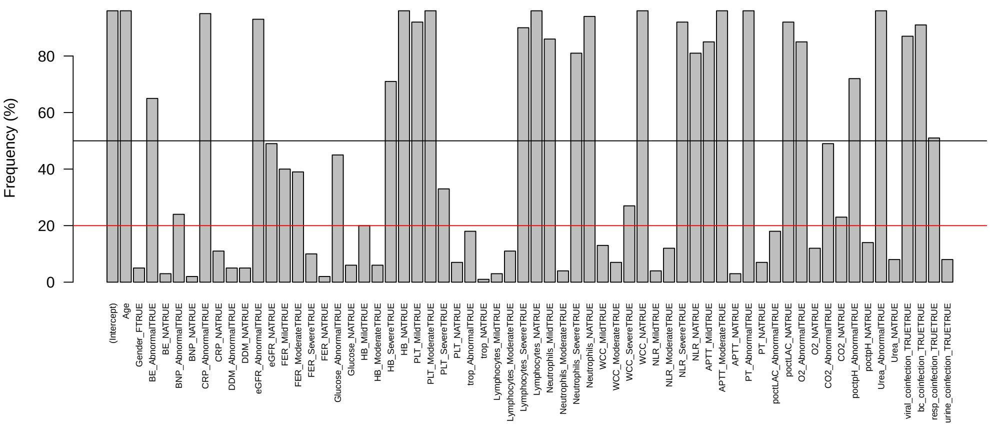

Fig. A10: Frequency of LASSO logistic regression variables having a coefficient greater or less than 0. Red and black lines indicate thresholds for 20% and 50% frequency respectively.

Solution Terms	AUC	AUC Standard Error	AUC Lower CI	AUC Upper CI	AUC Difference	AUC Difference Standard Error
(Intercept)	0.44	0.03	0.42	0.47	-0.35	0.04
**UreaAbnormal**	0.64	0.03	0.61	0.66	-0.16	0.02
HBNA	0.69	0.03	0.66	0.71	-0.11	0.02
**Age**	0.74	0.02	0.72	0.77	-0.05	0.02
poctLACNA	0.78	0.02	0.76	0.80	-0.02	0.01
**PTAbnormal**	0.79	0.02	0.78	0.81	0.00	0.01
NeutrophilsNA	0.79	0.02	0.78	0.81	0.00	0.01
**CRPAbnormal**	0.80	0.02	0.78	0.82	0.00	0.01
**NLRSevere**	0.81	0.02	0.79	0.83	0.01	0.01
eGFRNA	0.81	0.02	0.79	0.83	0.01	0.01
APTTModerate	0.81	0.02	0.79	0.83	0.01	0.01
PLTMild	0.81	0.02	0.79	0.83	0.01	0.01
PLTModerate	0.82	0.02	0.80	0.83	0.02	0.01
NeutrophilsMild	0.82	0.02	0.80	0.84	0.02	0.01
NeutrophilsSevere	0.82	0.02	0.80	0.84	0.02	0.01
LymphocytesSevere	0.82	0.02	0.80	0.84	0.02	0.01
bccoinfectionTRUE	0.82	0.02	0.80	0.84	0.02	0.01
HBSevere	0.82	0.02	0.80	0.84	0.02	0.01
poctpHAbnormal	0.82	0.02	0.80	0.84	0.02	0.00
eGFRAbnormal	0.82	0.02	0.80	0.84	0.02	0.00

Fig. A11: Summary statistics of Bayesian projective prediction ranking the contribution of each variable by change in AUC via 5-fold variable selection on full training data (n=534). Biomarkers in bold were selected for the reduced variable model (using all reading categories not just those listed.

Biomarker	Binary Categorical Variable	Bayesian Horshoe Odds Ratios CI [2.5%, 97.5%]	Projective Prediction 4-Biomarker Model Odds Ratios CI [2.5%, 97.5%]
Demographics / Other			
Age	-	1.05 [1.02, 1.07]	1.02 [1.01, 1.04]
Gender	Female	1.2 [0.71, 2.04]	
Blood Clotting Tests			
Activated partial thromboplastin time	Mild	1.82 [0.55, 6.03]	
	Moderate	4.9E+08 [0, Inf]	
	Not Taken	0.81 [0.12, 5.43]	
Prothrombin Time	Abnormal	3.76 [1.29, 10.91]	2.36 [1.06, 4.87]
	Not Taken	1.42 [0.21, 9.86]	0.75 [0.52, 1.03]
Blood Gas Tests			
Carbon Dioxide	Abnormal	0.83 [0.29, 2.42]	
	Not Taken	NA [NA, NA]	
Lactate	Abnormal	0.69 [0.14, 3.4]	
	Not Taken	0.28 [0.05, 1.64]	
Oxygen	Abnormal	1.79 [0.36, 8.93]	
	Not Taken	NA [NA, NA]	
Bicarbonate Excess	Abnormal	1.66 [0.85, 3.24]	
	Not Taken	0 [0, Inf]	
pH acid/base scale	Abnormal	1.22 [0.64, 2.33]	
	Not Taken	1.1E+07 [0, Inf]	
Coinfection			
Blood Culture	TRUE	0 [0, Inf]	
Respiratory	TRUE	4.59 [0.45, 46.35]	
Urine	TRUE	0.75 [0.11, 5.14]	
Viral	TRUE	11.37 [0.86, 150.44]	
Diabetes			
Glucose	Abnormal	1.78 [0.67, 4.74]	
	Not Taken	1.18 [0.59, 2.33]	
Full Blood Count Tests			
Hemoglobin	Mild	1.29 [0.73, 2.3]	
	Moderate	0.93 [0.37, 2.35]	
	Severe	4 [0.48, 32.96]	
	Not Taken	2.55E+18 [0, Inf]	
Platelet Count	Mild	2.86 [1.33, 6.11]	
	Moderate	5.97 [1.25, 28.56]	
	Severe	1.66 [0.02, 131.4]	
	Not Taken	0 [0, Inf]	
Lymphocytes	Mild	1.54 [0.63, 3.79]	
	Moderate	1.69 [0.62, 4.61]	
	Severe	3.07 [0.83, 11.36]	
	Not Taken	NA [NA, NA]	
Neutrophils	Mild	0.07 [0, 0.87]	
	Moderate	1.05 [0, 329.16]	
	Severe	1.46 [0.62, 3.43]	
	Not Taken	0 [0, Inf]	
Neutrophil - Lymphocyte Ratio	Mild	1.16 [0.48, 2.79]	1.19 [0.83, 1.77]
	Moderate	1.01 [0.32, 3.22]	1.56 [1.10, 2.60]
	Severe	1.59 [0.32, 7.9]	2.26 [1.30, 4.66]
	Not Taken	NA [NA, NA]	89.50 [11.55, 731.19]
White Cell Count	Mild	0.88 [0.31, 2.48]	
	Moderate	0.9 [0, 372.32]	
	Severe	1.23 [0.46, 3.33]	
	Not Taken	NA [NA, NA]	
Urea & Electrolytes Tests			
C-Reactive Protein	Abnormal	15.4 [1.66, 142.81]	4.37 [1.26, 21.29]
	Not Taken	2.46 [0.1, 62.87]	2.25 [0.99, 12.79]
Estimated Glomerular Filtration Rate	Abnormal	1.22 [0.51, 2.94]	
	Not Taken	0.3 [0.07, 1.23]	
Urea	Abnormal	2.83 [1.72, 4.65]	3.26 [1.96, 5.32]
	Not Taken	1.07 [0.56, 2.53]	0.75 [0.31, 1.88]
Investigatory Tests			
Brain / B-type natriuretic peptide	Abnormal	5.17 [0.29, 92.25]	
	Not Taken	3.92 [0.28, 54.02]	
D-Dimer	Abnormal	0.35 [0.05, 2.72]	
	Not Taken	0.6 [0.1, 3.45]	
Ferritin	Mild	5.05 [0.45, 56.98]	
	Moderate	2.17 [0.45, 10.47]	
	Severe	0.6 [0.03, 11.92]	
	Not Taken	1.15 [0.39, 3.42]	
Fibrinogen	Mild	1.32 [0.61, 8.49]	
	Severe	0.92 [0.26, 2.21]	
	Not Taken	0.87 [0.39, 1.37]	
Lactate dehydrogenase	Mild	1.29 [0.7, 5.43]	
	Moderate	1.07 [0.56, 2.51]	
	Severe	1.05 [0.47, 2.96]	
	Not Taken	0.71 [0.24, 1.22]	
Troponin-T	Abnormal	0.72 [0.19, 2.79]	
	Not Taken	0.7 [0.2, 2.46]	
Intercept			
Intercept	-	0.04 [0.0, 0.28]	0.01 [0.001, 0.03]

Fig. A12: Odds ratios for Bayesian model training on full training data (n=534) and a reduced 4-biomarker model via projective prediction

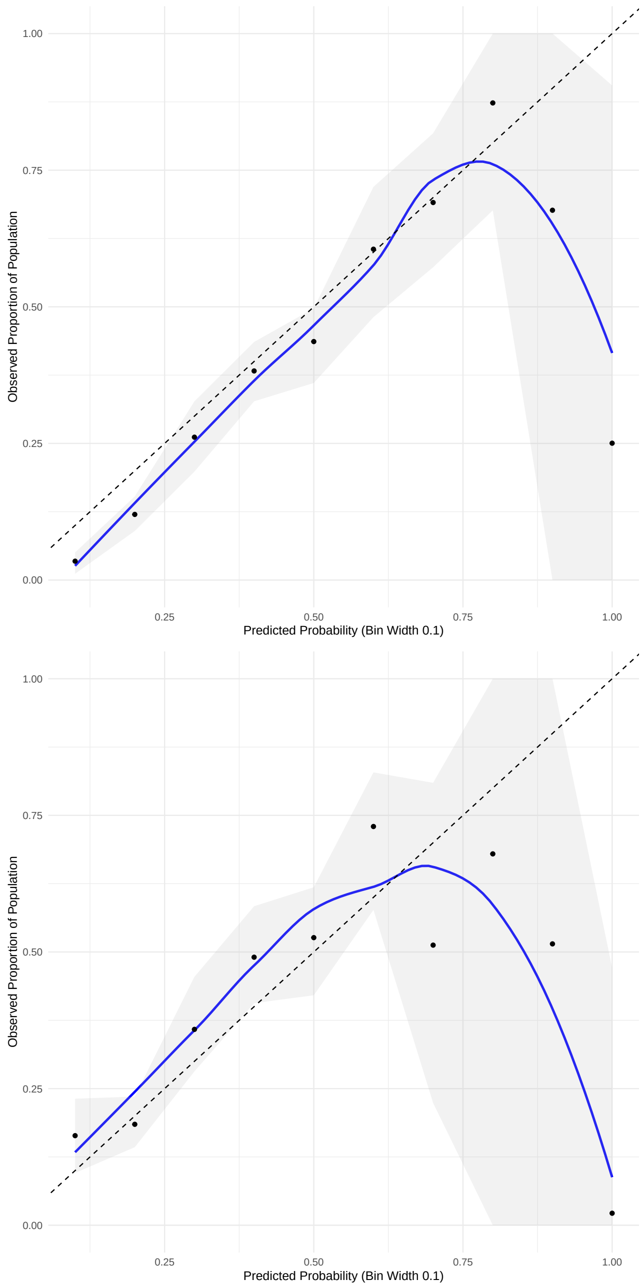

Fig. A13: Model calibration depicting Projective Prediction 4-biomarker model tested on: (Top) training data (n=534); (Bottom) validation data (n=222). The blue line is a loess smoothed curve across all cross-validation trials. Black circles represent the median value across trials. The grey shaded area indicates the 95% observed interval. Note the models do not have points for range 0-0.1 as no patients were in this band as judged by the model output. A well calibrated model should evenly distribute outcome probabilities, i.e. be close to unity.
